# Variation in Pediatric Anesthesiologist Sedation Practices for Pediatric Gastrointestinal Endoscopy

**DOI:** 10.3389/fped.2021.709433

**Published:** 2021-08-11

**Authors:** Kayla T. Hartjes, Tracey M. Dafonte, Austin F. Lee, Jenifer R. Lightdale

**Affiliations:** ^1^Division of Pediatric Gastroenterology, Hepatology and Nutrition, MassGeneral Hospital for Children, Boston, MA, United States; ^2^Department of Population and Quantitative Health Sciences, University of Massachusetts Medical School, Worcester, MA, United States; ^3^Division of Pediatric Gastroenterology and Nutrition, UMass Memorial Children's Medical Center, Department of Pediatrics, University of Massachusetts Medical School, Worcester, MA, United States

**Keywords:** sedation, endoscopy, anesthesiologist, pediatrics, variation in care, coefficient of variability, efficiency, pediatric anesthesiology

## Abstract

**Background:** Despite a worldwide shift toward anesthesiologist-administered sedation for gastrointestinal endoscopy in children, ideal sedation regimens remain unclear and best practices undefined.

**Aim:** The aim of our study was to document variation in anesthesiologist-administered sedation for pediatric endoscopy. Outcomes of interest included coefficients of variation, procedural efficiency, as well as adverse events.

**Methods:** IRB approval was obtained to review electronic health records of children undergoing routine endoscopy at our medical center during a recent calendar year. Descriptive and multivariate analyses were used to examine predictors of sedation practices.

**Results:** 258 healthy children [2–21 years (median 15, (Q1–Q3 = 10–17)] underwent either upper and/or lower endoscopies with sedation administered by anesthesiologists (*n* = 21), using different sedation regimens (29) that ranged from a single drug administered to 6 sedatives in combination. Most patients did not undergo endotracheal tube intubation for the procedure (208, 81%), and received propofol (255, 89%) either alone or in combination with other sedatives. A total of 10 (3.8%) adverse events (9 sedation related) were documented to occur. The coefficient of variation (CV) for sedation times was high at 64.2%, with regression analysis suggesting 8% was unexplained by procedure time. Multivariable model suggested that longer procedure time (*p* < 0.0001), younger age (*p* < 0.0001), and use of endotracheal tube intubation (*p* = 0.02) were associated with longer sedation time.

**Discussion:** We found great variation in anesthesiologist administered regimens for pediatric endoscopy at our institution that may be unwarranted, presenting may opportunities for minimizing patient risk, as well as for optimizing procedural efficiency.

## Key Points

1. To date, there is no single sedative or combined regimen that has been established as ideal for pediatric gastrointestinal procedures, regardless of whether procedural sedation is being administered by endoscopists or anesthesiologists.
2. Over the past two decades, pediatric endoscopy is increasingly being performed with anesthesiologist-administered sedation regimens that use propofol.
3. Broadly speaking, sedation plans that call for general anesthesia with endotracheal intubation are not necessary for routine pediatric endoscopy or colonoscopy and may decrease procedural efficiency and value.
4. It is becoming increasingly important for pediatric endoscopists to engage in a dialogue with anesthesiologists, with the goal of determining best sedation practices for children undergoing gastrointestinal procedures.

## Introduction

Over the past 2 decades, the landscape of sedation practices for pediatric endoscopy has shifted toward anesthesiologist-administration, despite no single sedative or regimen yet to be established as ideal (1–3). Historically, pediatric endoscopic sedation has been administered by endoscopists or anesthesiologists, and is generally considered necessary for children to undergo procedures (4). The trend toward anesthesiologist-administration has evolved from increasing interest in ensuring patient safety and comfort, (5, 6) as well as the ability of propofol to target a spectrum of sedation levels with rapid induction and recovery times (7). However, it is not clear that these and other anticipated benefits of anesthesiologist-administration for pediatric endoscopy have been fully realized, perhaps due to wide variations in care that have yet to be systematically documented or examined (1, 8–11).

Multiple studies have shown propofol, either as a total intravenous anesthetic (TIVA) or in combination with inhalational agents, to be highly effective for endoscopic sedation in children (12–16). Generally speaking, anesthesiologists differ from endoscopists in their regulatory license to use propofol and inhalational anesthetics, as well as to aim for deep levels of sedation or general anesthesia (17, 18). Anesthesiologists may therefore be more equipped to administer sedation regimens that can assure children will tolerate endoscopic procedures, without exhibiting agitation, vocalization and disruptive movements (6). Nevertheless, anesthesiologist-administration has not decreased the occurrence of sedation related adverse events in children undergoing upper and lower endoscopy (19, 20). Adverse events associated with sedation, such as apnea, laryngospasm and bradycardia- even when sedation practices involve anesthesiologists - continue to occur more often during pediatric endoscopy than procedural complications, such as mucosal bleeding or perforation (1, 12, 19–22).

Another important concern regarding use of anesthesiologists during pediatric endoscopy is the potential for inefficient use of healthcare resources (2, 8, 9, 23). For example, unnecessary use of endotracheal intubation for routine diagnostic endoscopy in children has been shown to increase endoscopy room times and costs (11). While many endoscopists acknowledge increased patient comfort when anesthesiologists provide sedation, it has also been true that variability in anesthesiologist practices can lead to a mismatch between sedation provided and the procedure performed (8). For example, provider variation in the use of rapid sequence intubation has been associated with much longer sedation times relative to procedural duration, as well as patient paralysis when immobility is not required for endoscopy (24). Ultimately, reducing unwarranted variation in pediatric anesthesiologist sedation for endoscopy will likely be necessary to improve patient safety, as well as to ensure procedural efficiency and value (19).

We believe the intersection between gastrointestinal procedures in children, patient safety, efficiency and sedation regimens remains of great importance to study in the current era of anesthesiologist-administration – particularly because unwarranted variation in anesthesiology sedation practices has been speculated to exist and best practices have yet to be identified (1, 12). As a first step in examining this topic, we undertook to systematically document variation in anesthesiology sedation practices for pediatric endoscopy at our institution. We were specifically interested in examining how various sedative regimens, anesthesiology and endoscopy providers, provider staffing models, and use of endotracheal intubation might interrelate with procedure and patient factors, including procedure type, age, and medical complexity. Outcomes of interest included sedation and procedural efficiency, as well as both sedation and non-sedation related adverse events.

## Methods

Institutional approval (Protocol # H00013675) was granted to develop and analyze a complete retrospective database of all endoscopic procedures performed by pediatric gastroenterologists with anesthesiologist-administered sedation at our academic medical center during calendar year 2018. An endoscopy reporting database (ProVation MD) was used to identify all children who underwent upper and/or lower endoscopic procedures performed during the study period for routine, diagnostic purposes. Two independent investigators (KH, TD) codified and abstracted information about each case from components of the electronic medical record (Epic) onto an institutionally approved separate case report form, including from the endoscopists' procedure reports, endoscopy technician and nurse peri-procedure documentation forms, as well as from the anesthesiologists' records. Patient descriptive data was recorded, including sex, age, height, weight, medication allergies and American Society of Anesthesiology (ASA) patient complexity status, as documented by the anesthesiologist. Type of procedure performed (upper endoscopy, lower endoscopy, both upper and lower endoscopy), time first sedative administered, time out of room, time of endoscope insertion, and time of endoscope removal were recorded, as well as the indication for the procedure, whether the patient underwent endotracheal intubation as part of the sedation plan with or without paralytic agents, and/or documented adverse events. We also noted the names and doses for all oral and intravenous sedatives that were administered during the sedation time, including midazolam, fentanyl, propofol, ketamine; as well as names of all inhalational anesthetics, including sevoflurane, isoflurane, and nitrous oxide. In addition, we recorded and coded the identities of all endoscopists who performed procedures, anesthesiologists of record for administration of the sedation, and any certified registered nurse anesthetists (CRNAs) who were documented to have delivered sedation with anesthesiologist supervision, during each case.

The first sedative administered was defined as any oral, intravenous (IV) or inhalational agent administered for the purposes of anxiolysis, analgesia or inducing sedation, and included oral midazolam if administered in the pre-operative area for anxiolysis prior to transport to the endoscopy room. We excluded any sedatives administered in the recovery area for agitation, delirium, or other adverse sedation events, although these events were recorded as below. We defined sedation time as first sedative administered to patient time out of room, and procedure time as scope in to scope out.

Adverse events are predefined at our institution and include apnea, disordered respiration, laryngospasm, vomiting, aspiration, delirium, agitation, inadequate sedation for a procedure, as well as airway management issues, intravenous line infiltration, patient pain or discomfort, bleeding, procedural complications, unanticipated admission to the hospital, and death. Any adverse event that was recorded as such in the endoscopy report or the anesthesia record was abstracted to the study case report form. For the purposes of analysis, adverse events were categorized to be either sedation-related or other.

### Patients and Procedures

We included all patients ages 1–21 years old who underwent routine, diagnostic upper and/or lower endoscopy at UMASS Medical Center with anesthesiologist-administered sedation during the study period. We excluded pregnant patients, as well as patients undergoing emergency or add-on procedures, including for gastrointestinal bleeding, foreign body, or caustic ingestions. We also excluded patients undergoing procedures that were performed in combination under the same sedation with non-gastrointestinal procedures performed by other subspecialists – including otolaryngologists performing laryngoscopies or pulmonologists performing bronchoscopies. We also excluded procedures that involved endoscopic interventions, including dilations and polypectomies. All endoscopic procedures were performed in a hospital-based operating room setting by an American Board of Pediatric (ABP) certified pediatric gastroenterologist attending. All patients received anesthesiologist-administered sedation regimens that was either provided or overseen by an attending anesthesiologist with pediatric training, who at times was assigned a CRNA to assist in providing sedation care. The targeted depth level of sedation for all patients was at least deep sedation. We defined all patients who underwent endotracheal intubation in our study to have received general anesthesia.

### Study Outcomes

Our primary outcome of interest was variation in sedation regimens. We sought to characterize this in terms of sedative names and types (oral vs. IV vs. inhalational), as well as number of sedatives employed. Secondary outcomes included total anesthesiologist- administered sedation time, and sedation or non-sedation related adverse events.

### Statistical Analysis

We described continuous variables representing provider experience (e.g., number of endoscopist sedations administered during the study period; number of procedures performed during the study period), patient's characteristics (e.g., age, weight), sedation (e.g., sedative doses), and procedure characteristics (e.g., anesthesiologist-administered sedation time, procedure time) using medians, lower (Q1) and upper (Q3) quartiles. Categorical variables, including whether patients underwent endotracheal intubation or experienced an adverse event, were tabulated using proportions. Box and whisker plot was drawn to display the distribution of anesthesiologist-administered sedation time by procedure type. To identify predictors for length of anesthesiologist-administered sedation time, we performed multivariable generalized linear regression model with potential predictors including patient's age and sex, ASA level, length of procedure, procedure type, adverse events, and use of endotracheal intubation and CRNA. To identify predictors for use of endotracheal intubation, which was dichotomized as yes vs. no, we performed multivariable logistic regression model which yielded odds ratio (OR) of using the tube for each predictor. Both regression models were incorporated with generalized estimating equation (GEE) to account for potential correlations between repeated measures by anesthesiologists. Statistical analyses were performed using SAS v9.4 (SAS Institute, NC) and S-Plus 7 for Windows (Insightful, USA).

## Results

**Figure 1:**
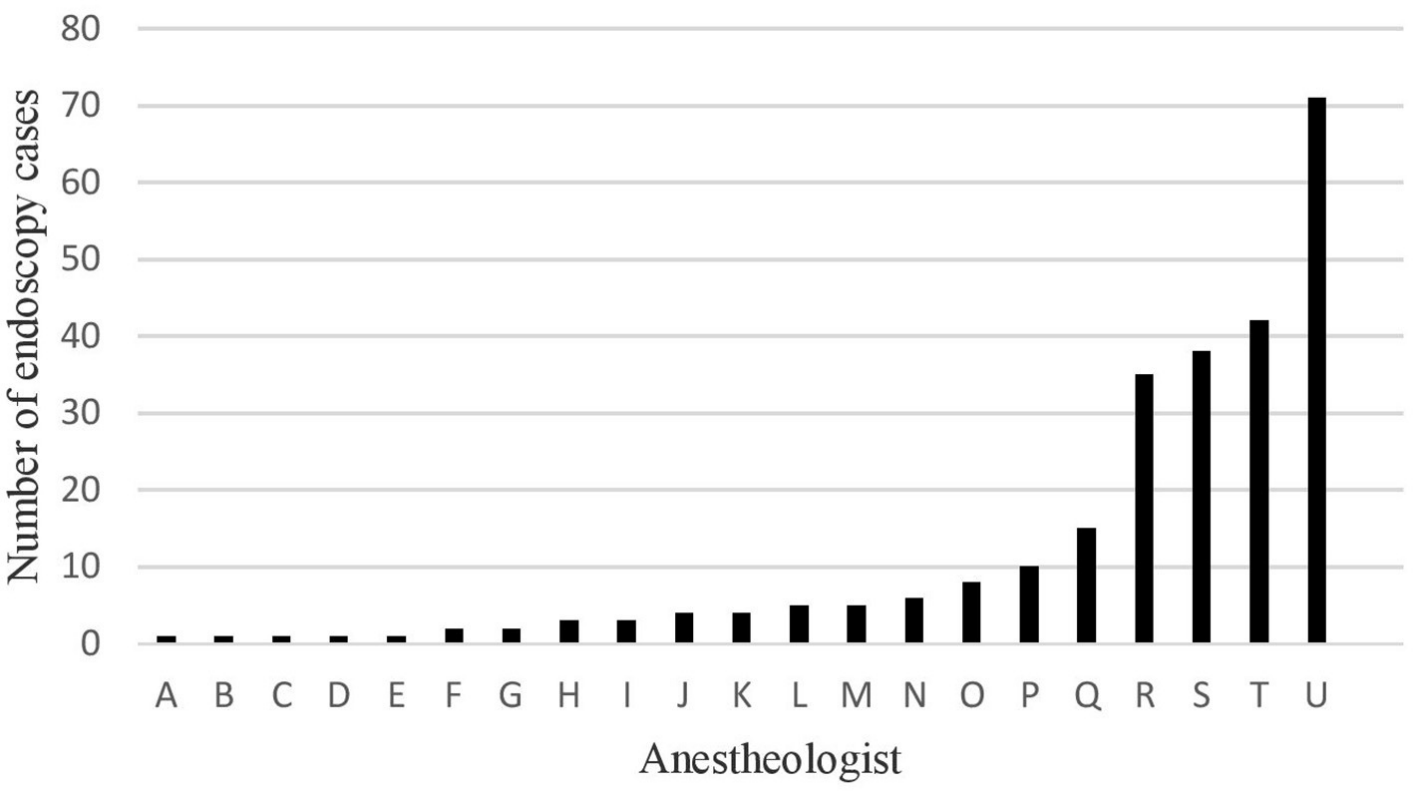
Number of endoscopy cases performed by each anestheologist (*N* = 21).

A total of 258 upper and lower routine, diagnostic, endoscopic procedures were performed at our institution in patients ages 2–20 years of age with median age of 15 years (Q1-Q3 = 10–17, Table 1). Most patients were older than 9 years of age (*n* = 197, 76%), and were healthy with an ASA status of ≤ 2 (249, 97%). The number of cases that each anesthesiologist (*n* = 21) staffed during the study period varied widely, ranging from 1–71 (Figure 1). Anesthesiologists were assigned a certified registered nurse anesthetist (CRNA) to work with them for 205 (80%) of the cases. Most anesthesiologists (11, 52%), and CRNAs (15, 60%) provided sedation care for <5 endoscopic cases over the study period. Five anesthesiologists administered endoscopic sedation for a single case each, while 4 staffed at least 30 cases over the calendar year.

**Table 1:** Descriptive characteristics of patients, providers and procedures.

	**Summary statistics**
**Patients (** ***N*** **=** **258)**	
Age (years), *median (Q1, Q3)*	15 (10, 17)
Weight (Kg), *median (Q1,Q3)*	52.3 (35, 63)
Gender (male), *n* (*%)*	126 (49)
ASA, *n (%)*	
ASA 1	77 (30)
ASA 2	172 (67)
ASA 3	8 (3)
ASA 4	1 (<1%)
ETT intubation for sedation, *n (%)*	50 (19)
**Providers**	
Total number of endoscopists, *n*	6
Total number of endoscopies per	32 (20.3, 60.3)
Endoscopist, *Median (Q1, Q3)*	
Total number of attending anesthesiologists, n	21
Total number of cases per anesthesiologist,	4 (1.5, 12.5)
*Median (Q1, Q3)*	
Total number of CRNAs, n	26
Total number of cases per CRNA,	3.5 (1,11.25)
*Median, (Q1, Q3)*	
**Procedures (** ***N*** **=** **258)**	
Procedure time (minutes), *median (Q1,Q3)*	14 (8, 35)
Anesthetic Time (minutes), *median (Q1,Q3)*	25 (17, 41)
Type of Procedure, *n* (%)	
Upper endoscopy	147 (57)
Lower endoscopy	28 (11)
Upper and lower endoscopy	83 (33)
Procedure Indication, *n* (%)	
Abdominal pain	85 (33)
Positive celiac serologies	34 (13)
Hematochezia	25 (10)
Diarrhea	18 (7)
Reflux symptoms	23 (9)
Known inflammatory bowel disease	22 (9)
Dysphagia	21 (8)
Known Eosinophilic esophagitis	16 (6)
Other	14 (5)
Adverse events, *n* (%)	
Sedation-related	9 (3.5)
Other	1 (<1)

*Q1 = 25th percentile; Q3 = 75th percentile; ASA, American Society of Anesthesiology (ASA) Patient Classification; ETT, endotracheal tube*.

*Mean anesthetic time = 31.8 mins, SD = 20.4, coefficient of variation (CV) = 64.2%*.

### Sedation Practices

A total of 29 sedation regimens, ranging from a single drug administered to 6 sedatives in combination, were administered to patients at our institutions during the study period (Table 2). Most patients (*n* = 192, 74%) did not receive pre-operative oral midazolam or undergo endotracheal intubation for the procedure (208, 81%). Patients who underwent endotracheal intubation had a greater number of sedative agents administered during cases, with 15/50 (30%) patients who had endotracheal intubation receiving a regimen that involved ≥5, compared with 7/208 (3%) of patients who were not intubated for the procedure (*p* < 0.0001 by Chi-square test of proportions).

**Table 2:** Descriptive information about sedatives, as well as single and combination drug regimens, including frequency of use and PO/IV doses used.

**Sedative**	**N^**°**^ of Patients/Cases (%) (*N* = 258)**	**Dose Median (Q1, Q3)**
Fentanyl (mcg/kg)	100 (39)	0.823 (0.48, 1.24)
Ketamine (mg/kg)	3 (1)	0.21 (0.16, 0.67)
Midazolam PO (mg/kg)	67 (26)	0.39 (0.32, 0.49)
Midazolam IV (mg/kg)	117 (45)	0.033 (0.03, 0.04)
Propofol (mg/kg)	255 (89)	5.53 (3.64, 9.35)
Nitrous Oxide	112 (43)	–
Sevoflurane	122 (47)	–
Isoflurane	2 (<1)	–
**Sedative Regimens**		
**Single Drug**, ***n*****(%)**	**13 (5)**	
Propofol only	12 (4.5)	10.3 (5.84, 13.31)
Midazolam IV	1 (<1%)	0.03
**Double drug**, ***n*****(%)**	**66 (26)**	
Propofol + Fentanyl	5 (2)	4.58 (3.5, 11.97) + 0.75(0.48, 1.51)
Propofol + Midazolam PO	6 (2)	8.83 (6.89, 10.9) + 0.27(0.13, 0.38)
Propofol + Midazolam IV	47 (18)	5.94 (4.53, 11.76) + 0.03 (0.03, 0.04)
Propofol + Nitrous Oxide	3 (1)	6.8 (6.49, 7.39) + IA
Propofol + Sevoflurane	4 (2)	7.93 (6.25, 9.41) + IA
Other^1^	1 (<1)	
**Triple drug**, ***n*****(%)**	**106 (41)**	
Propofol + Midazolam IV + Fentanyl	42 (16)	6.74 (4.28, 10.44) + 0.035 (0.03, 0.04) + 0.62 (0.45, 0.89)
Propofol + Nitrous oxide + Sevoflurane	39 (15)	5.12 (3.57, 10.48) + IA + IA
Propofol + Midazolam PO + Sevoflurane	6 (3)	4.47 (3.62, 7.54) + 0.25 (0.70, 0.52) + IA
Propofol + Midazolam PO + Fentanyl	8 (3)	5.15 (3.03, 12.16)+ 0.03 (0.03, 0.38) + 0.90 (0.4, 1.14)
Propofol + Midazolam PO + Nitrous oxide	5 (2)	11.53 (4.35 + 13.15)+ 0.29 (0.23, 0.42) + IA
Other^1^	6 (2)	
**Quadruple drug**, ***n*****(%)**	**51 (20)**	
Propofol + Midazolam PO + Nitrous oxide + Sevoflurane	25 (10)	3.75 (2.8 + 5.7) + 0.45 (0.36, 0.50) + IA + IA
Propofol + Fentanyl +Nitrous oxide + Sevoflurane	10 (4)	3.93 (1.94, 7.41) + 0.72 (0.53 + 1.1) + IA + IA
Propofol + Midazolam IV + Fentanyl + Sevoflurane	6 (2)	3.5 (2.69, 4.31) + 0.03 (0.02 + 0.04) + 0.89 (0.64, 1.77) + IA
Other^1^	10 (4)	
**Five drugs or more**, ***n*****(%)**^**1**^	**22 (9)**	

*Q1 = 25th percentile; Q3 = 75th percentile; IV, intravenous; PO, oral; IA, inhalational anesthetic*.

1 *The other double drug regimen was midazolam IV + fentanyl (1 case). Other triple drug regimens included midazolam IV + propofol and either nitrous oxide (2 cases) or sevoflurane (1 case) or ketamine (1 case). Another triple drug regimen consitented of midazolam IV + fentanyl and sevofluance (1 case) and another regimen of propofol + fentanyl + nitrous oxide (1 case). Other quadruple drug regimens included propofol + sevoflurane in combination with either nitrous oxide + midazolam IV (4 cases) or midazolam PO + fentanyl (4 cases) or midazolam PO + ketamine (1 case); and nitrous oxide + midazolam PO + fentanyl + propofol (1 case). Five drug regimens include: nitrous oxide + sevoflurane + fentanyl + propofol in combination with either midazolam IV (9 cases) or midazolam PO (11 cases). Six drug regimens included: nitrous oxide + isoflurane + midazolam IV + fentanyl + propofol with either sevoflurane (1 case) or ketamine (1 case)*.

Most patients (255, 89%) received infused propofol either alone or in combination with other medications, with the most common regimen (TIVA with propofol and midazolam) used in 47 (18%) patients. Among patients who did not receive propofol, 1 received an infusion of midazolam as a single drug regimen, and 2 received sevoflurane with intravenous midazolam and fentanyl. Use of inhalational anesthetics also varied, with many patients receiving more than 1 volatile gas during the case.

### Adverse Events

A total of 10 (3.8%) institutionally defined adverse events were documented to occur. These included 9 that were categorized for study purposes as related to sedation [bradycardia (1), laryngospasm (3), inappropriately woke during procedure (1), use of reversal agent (1), post-op delirium (2), and stridor (1)], as well as 1 adverse event (IV infiltration) that was categorized for study purposes as not related to sedation. We could not find any pattern regarding patient demographics, procedure characteristics and sedation regimens among patients who experienced adverse events (data not shown), although endotracheal intubation in univariate analysis was noted to be weakly associated with adverse events (OR 2.93, 95% CI: 0.89, 9.60, *p* = 0.08). By grouping anesthesiologists into those who staffed >5 endoscopic cases vs. those ≤5 during the study period, mean number of adverse events between two groups was not significantly different (*p* = 0.23 by two-sample *t* test).

### Efficiency

**Figure 2:**
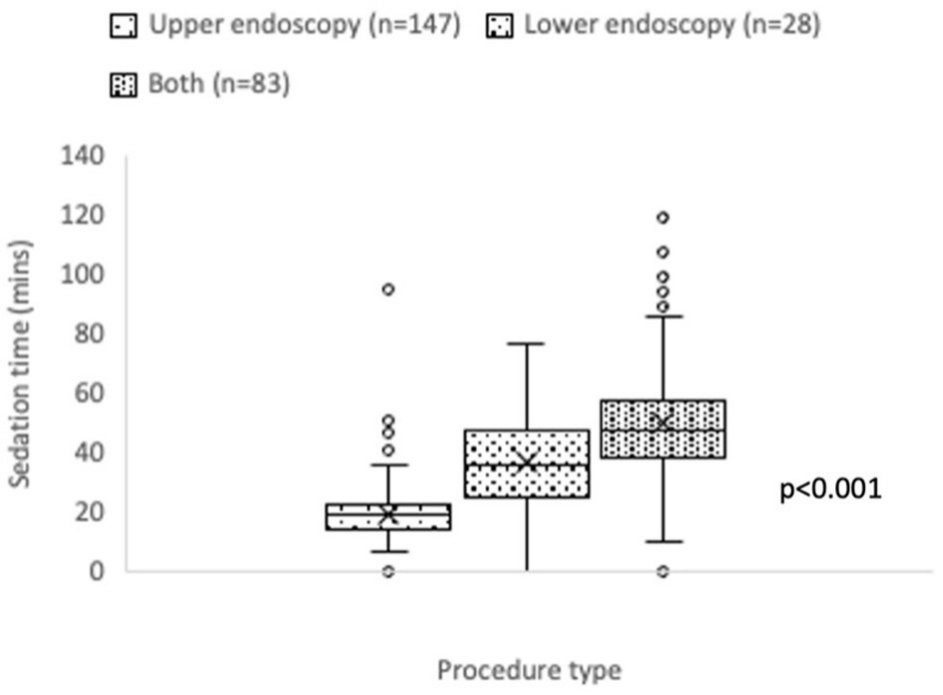
Sedation time (minutes) by procedure type.

Mean anesthesiologist-administered sedation time was 31.8 (±20.4 SD) minutes, with median 25 (17–41) minutes. The coefficient of variation (CV) for sedation time was 64.2%, indicating a wide variation across all procedures. Highly skewed sedation time was seen for upper endoscopic procedures, and for combined upper and lower endoscopic procedures (Figure 2).

**Figure 3:**
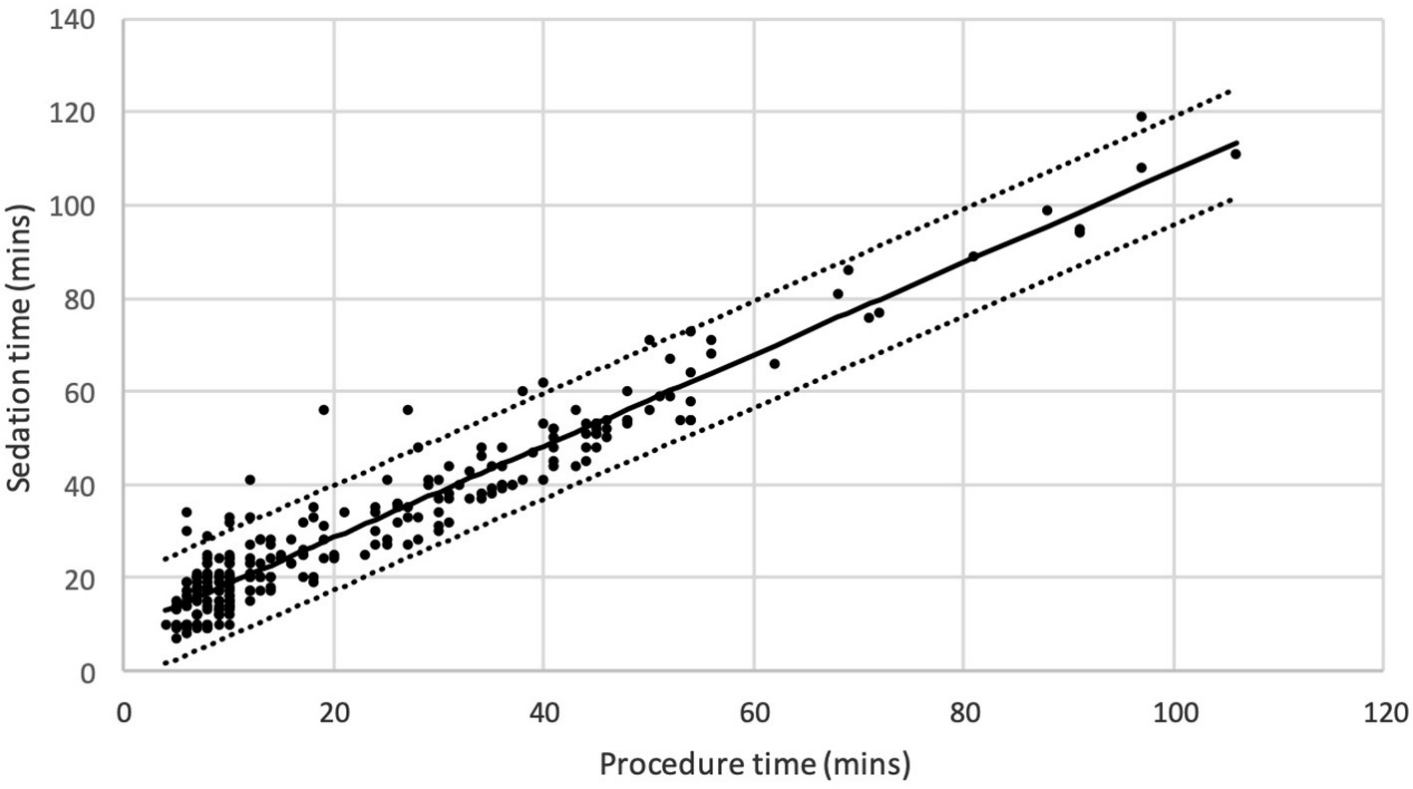
Scatter plot for sedation time vs. procedure time with prediction line and 95% prediction limits (*N* = 258, R-square = 92%).

Furthermore, regression analysis showed that 92% of variation in anesthesiologist-administered sedation time was explained by procedure time (Figure 3). In other words, 8% was unexplained by procedure time.

Multivariable model suggested that longer procedure time (*p* < 0.0001), younger age (*p* < 0.0001), and use of endotracheal intubation (*p* = 0.02) were associated with longer sedation time (Table 3). Endotracheal tube intubation was performed for procedures in 50 (19%) patients and was associated with longer sedation time (OR = 1.02, 95% CL 1.00–1.04, *p* = 0.04) and patient's younger age (OR = 0.93, 95% CL 0.88–0.98, *p* = 0.0047), higher ASA level (level 2 OR = 1.90, *p* < 0.0001, and level 3&4 OR = 3.61, *p* = 0.005, respectively when compared to level 1, Table 4). Endotracheal tube intubation also varied by procedure type. When compared to lower endoscopic procedures, upper procedures were more than 3 times likely to have patients undergo endotracheal tube intubation (OR = 4.33, 95% CL 2.23–8.44, *p* < 0.0001).

**Table 3:** Multivariate normal regression model on sedation time (mins).

**Predictor**	**Category**	**Estimate**	**SE**	**95% Confidence Limits**		***p* value**
Age (years)	Continuous	−0.53	0.09	−0.70	−0.36	<0.0001
Male	Vs female	0.63	0.74	−0.81	2.08	0.39
ASA level	2 vs. 1	0.0	0.7	−1.4	1.3	0.95
	3 & 4 vs. 1	3.8	2.3	−0.7	8.3	0.10
Procedure time (mins)	Continuous	0.98	0.03	0.93	1.03	<0.0001
Procedure type	Lower endoscopy vs. Upper endoscopy	0.2	1.2	−2.2	2.6	0.88
	Both vs. Upper endoscopy	2.2	1.3	−0.4	4.7	0.10
Adverse events	Yes vs. No	3.1	2.0	−0.9	7.0	0.13
Endotracheal intubation	Yes vs. No	1.8	0.8	0.2	3.3	0.0229
CRNA	Yes vs. No	−1.3	1.3	−3.8	1.2	0.31

*Total procedure cases = 258, performed by 21 anesthesiologists*.

*The model was multivariate normal regression, incorporated with generalized estimating equation (GEE) to account for potential correlations between repeapted measures by anesthesiologists*.

**Table 4:** Multivariate logistic regression model on endotracheal tube intubation.

**Predictor**	**Category**	**OR**	**95% Confidence Limits**		***p* value**
Age (years)	Continuous	0.93	0.88	0.98	0.0047
Male	Vs. female	1.25	0.92	1.69	0.15
ASA level	2 vs. 1	1.90	1.43	2.51	<0.0001
	3 & 4 vs. 1	3.61	1.47	8.85	0.0050
Sedation time (mins)	Continuous	1.02	1.00	1.04	0.0413
Procedure type	Upper vs. lower endoscopy	4.33	2.23	8.44	<0.0001
	Upper & lower vs. lower endoscopy	3.33	2.20	5.04	<0.0001
Adverse events	Yes vs. no	1.95	0.59	6.50	0.27
CRNA	Yes vs. no	1.12	0.64	1.98	0.69

*Total procedure cases = 258, performed by 21 anesthesiologists*.

*The model was multivariate logistic regression, incorporated with generalized estimating equation (GEE) to account for potential correlations between repeapted measures by anesthesiologists*.

## Discussion

The results of our study show great variation in sedation regimens used by staff anesthesiologists caring for children undergoing gastrointestinal endoscopy at our hospital. Indeed, so many drugs were used in different combinations, it was difficult to determine any predictive factors or patterns and no dominant regimen was identified. We believe this variation in anesthesiologist-administered regimens for pediatric endoscopy reflects a paucity of evidence-based or consensus best practices that leads anesthesiologists at our institution and elsewhere to determine their own preferences.

Our results suggest provider-driven variation may have an impact on quality and safety outcomes. While procedural times were a primary factor in variation of sedation efficiency, about 8% of variation remained unexplained. This may be particularly the case for upper endoscopy, where provider decision to perform endotracheal intubation may affect procedural efficiency. Although multivariate analysis of our single-institution sample suggests some association for sedation decision making around endotracheal intubation with procedure type and patient characteristics, the dramatic spectrum of sedation practices that was documented across anesthesiology providers raises the specter that at least some variation in anesthesiologist sedation practices may be unwarranted. Our results also affirm that adverse events occur with anesthesiologist-administered sedation more commonly than non-sedation related events and continues to suggest that even when anesthesiologists are administering the sedation, improving child safety during endoscopy is highly dependent upon seeking improvements in sedation regimens.

We suspect the magnitude of variation in sedation protocols used at any institution likely reflects local preferences and the number of anesthesiologists who may be involved with staffing cases (25). Few guidelines exist that address anesthesiologist-administered sedation for pediatric gastrointestinal procedures, (2, 18, 25) and none directly identify regimens that may be ideal. All agree that the primary purpose of sedation for children undergoing upper and lower endoscopies is to perform procedures safely, with a minimal amount of emotional and physical discomfort. Although many sedatives have been shown to be safe and effective for endoscopic sedation, all have the potential to significantly depress the central nervous system, airway protective reflexes, and ventilation (1, 12, 26). Those with narrow therapeutic windows such as propofol may be even more likely to be associated respiratory events (7, 15). Kaddu et al. reported that 20% of pediatric patients receiving anesthesiologist administered propofol for upper endoscopy experienced transient apnea (14).

Rates of adverse events in our study mirror those published for endoscopist-administered sedation for pediatrics (14, 25), as well as for anesthesiologist administered rates at other institutions (19, 26, 27). In terms of safety, both the American Society of Gastrointestinal Endoscopy (ASGE) (2) and the American Academy of Pediatrics (AAP) (25) advise tailoring sedation plans according to a patient's physical status, as classified by the American Society of Anesthesiology (ASA). (28) Considering a patient's age and developmental status may also be of importance. Larger studies have suggested that generally speaking, the smallest and youngest pediatric patients with the highest ASA classifications are at greatest risk for complications during gastrointestinal procedures (19, 21, 22).

Adverse events and prolonged sedations are more common with deeper levels, which are considered to stretch along a continuum without clear boundaries and are defined by a patient's response to verbal, light tactile or painful stimuli, as well as their vital signs (29). Deep sedation implies a medically controlled state of depressed consciousness from which the patient is not easily aroused but may respond purposefully to painful stimulation. General anesthesia describes the deepest level of sedation where the patient is unconscious, with reduced responses to stimuli, and with an airway that may require support. Of course, optimal levels of sedation may vary depending upon the procedure, and may be tricky to maintain during routine maneuvers intrinsic to endoscopy that can affect the fine line between lighter and deeper levels of sedation (1, 30). In upper endoscopy, a major overall goal of sedation may be to avoid gagging and increase patient cooperation, and it is reasonable to anticipate that the few seconds it takes to insert the endoscope will typically be the most stimulating part of the procedure, while colonoscopy sedation planning should anticipate visceral pain associated with looping (31). It is important to know that deep sedation may develop during patients undergoing longer procedures (i.e., combined endoscopy and colonoscopy), or after a decrease in painful stimuli (i.e., after successful navigation of the hepatic flexure) (30).

In our study, patients who underwent endotracheal intubation for the procedure had more sedatives given in combination regimens and had more adverse events. These results are also unsurprising. Patients who receive multiple doses and/or different sedatives have been shown to be at increased risk for deeper sedation than planned, and may be more likely to have adverse events (6). Child anxiety levels can also affect sedation and have been demonstrated to be reduced in randomized controlled trials of pre-operative medication with oral midazolam (6, 25). We noted <20% of patients at our institution received a regimen that included this evidenced-based approach to improving patient satisfaction and tolerability (27, 32).

In 2002, Wennberg defined “unwarranted variations” in care as those that cannot be explained by patient factors, including illness severity, indication for treatment or patient preference (33). More recent publications have examined variation among anesthesiologists terms of regional differences, as well as “professional uncertainty,” (34, 35) which may both contribute to variation in anesthesiologist sedation regimens for pediatric endoscopy. Our study was limited to a single institution. Ideally, future studies will examine how the variation we found at our relatively small children's medical center within a larger university hospital compares with similarly sized groups of either anesthesiologists or endoscopists, or with variation that may happen at larger children's hospitals with higher volume of pediatric endoscopy.

Our study was further limited by its design as a retrospective review of electronic medical records, which precluded a prospective understanding of variation in sedation practices which may have been warranted. We did not prospectively survey our anesthesiologists as they planned sedation regimens pre-operatively, nor collect data on why or how sedation plans may have been adjusted once the procedure was underway. Furthermore, the retrospective nature of our investigation also ensured heterogeneity in our patient population (i.e., a wide variety of patient ages), and a lack of comparative data so that we are unable to comment on benefits and risks of specific regimens that were used. Fortunately, we did find the range and mean ages of children undergoing endoscopy at our center to be similar that reported in other multicenter studies that have examined outcomes of endoscopy and sedation (21, 22), which lends credence to the generalizability of our population to pediatric gastroenterology centers.

Nevertheless, we believe the variation in sedation practices found in our study resulted from uneven anesthesiologist familiarity with upper and lower endoscopy as brief, non-surgical procedures that do not require strict patient immobility. While some anesthesiologists may specialize in endoscopic sedation for children and have developed preferred regimens, others may only be asked to provide it on rare occasions. Our study was not powered to examine patient safety, and our data did not show an inverse relationship between the number of anesthesiologist cases in our database and adverse events, nor with variation in sedation time. From our perspective as pediatric gastroenterologists, it is reasonable to assume that anesthesiologist familiarity with endoscopy is desirable.

We also believe anesthesiologist familiarity with routine gastrointestinal procedures in children is likely associated with more confidence in recognizing that it is possible, and even preferable, to employ a propofol-based regimen for most pediatric endoscopy without performing endotracheal intubation. The practice of avoiding unnecessary endotracheal intubation may be important to assuring secondary and desirable goals of endoscopic sedation, including maximizing procedural efficiency, minimizing recovery times, and maintaining cost-effectiveness (1, 12, 15, 25, 28). Although it has been suggested that shorter induction times associated with propofol should lead to improved procedural efficiency in pediatric endoscopy units, variations in anesthesiology practices may explain why this has not been found to be true (1, 8, 12, 23). Currently, routine endotracheal intubation of all children undergoing upper GI procedures is not supported in the anesthesia literature (23). It is also important to recognize that there is no consensus for medical indications or an age cut-off, and that the decision to intubate pediatric patients should be weighed against issues that may occur with instrumenting the airway, as well as with increasing depth and prolonging sedation time unnecessarily (36).

In our study, patients were less likely to undergo endotracheal intubation for colonoscopy. This was expected as a spontaneously breathing, propofol based regimen is particularly considered to be well suited for colonoscopy, where the risk of airway compromise is greatly reduced compared to upper endoscopy that stimulates the airway (37). On the other hand, propofol does not have analgesic properties and loop formation of the scope as well as maneuvers performed to reduce this (i.e., the application of external abdominal pressure) may cause pain and patient movement, leading to increased sedation requirements (38). As was seen in the few (~5%) patients in our study that received such a regimen, higher doses of a single-drug propofol TIVA may ensue, which in turn can increase patient risks (39, 40). Future studies should focus on identifying best practices for balancing propofol with analgesics for pediatric colonoscopy.

In conclusion, we believe the findings of our study contribute to the literature by illustrating striking variation in anesthesiologist-provided sedation care for children undergoing gastrointestinal endoscopy that likely extends beyond our institution to many others. In this way, our findings provide a mandate for all pediatric gastroenterologists to engage in a dialogue with our anesthesiology colleagues about the need to identify best practices for endoscopy sedation. While it has become standard in many ways for endoscopic sedation in children to administered by anesthesiologists, the number, doses, and combinations of sedatives may vary greatly, as does the use of endotracheal tube intubation. Unwarranted provider variation may explain why the trend toward anesthesiologist-administered sedation has not necessarily reduced the rate of adverse events related to sedation for endoscopy or improved procedural efficiency. Moving forward, we call upon all anesthesiologists who are providing endoscopic sedation for children to ensure that they are knowledgeable about routine gastrointestinal procedures, and that they are actively seeking to avoid unwarranted variations in care.

## Data Availability Statement

The raw data supporting the conclusions of this article will be made available by the authors, without undue reservation.

## Ethics Statement

The studies involving human participants were reviewed and approved by H00013675. Written informed consent from the participants' legal guardian/next of kin was not required to participate in this study in accordance with the national legislation and the institutional requirements.

## Author Contributions

JL: responsibility for the integrity of the data, accuracy of the data analysis, and writing first draft of the manuscript. JL, TD, KH, and AL: study concept and design, interpretation of data, critical revision of the manuscript for important intellectual content, and approval of final version. AL: analysis. All authors contributed to the article and approved the submitted version.

## Conflict of Interest

The authors declare that the research was conducted in the absence of any commercial or financial relationships that could be construed as a potential conflict of interest.

## Publisher's Note

All claims expressed in this article are solely those of the authors and do not necessarily represent those of their affiliated organizations, or those of the publisher, the editors and the reviewers. Any product that may be evaluated in this article, or claim that may be made by its manufacturer, is not guaranteed or endorsed by the publisher.

## Abbreviations

GI: Gastrointestinal
IV: Intravenous
ASA: American Society of Anesthesiology.

## References

[1] Chung HK Lightdale JR. Sedation and monitoring in the pediatric patient during gastrointestinal endoscopy. Gastrointest Endosc Clin N Am. (2016) 26:507–25. 10.1016/j.giec.2016.02.004 27372774

[2] Early DS Lightdale JR Vargo JJ Acosta RD Chandrasekhara V Chathadi KV. Guidelines for sedation and anesthesia in GI endoscopy. Gastrointest Endosc. (2018) 87:327–37. 10.1016/j.gie.2017.07.018 29306520

[3] Institute AG Vargo JJ DeLegge MH Feld AD Gerstenberger PD Kwo PY. Multisociety sedation curriculum for gastrointestinal endoscopy. Am J Gastroenterol. (2012) 143:e18–41. 10.1038/ajg.2012.112 22624720

[4] Lightdale JR Mahoney LB Schwarz SM Liacouras CA. Methods of sedation in pediatric endoscopy: a survey of NASPGHAN members. J Pediatr Gastroenterol Nutr. (2007) 45:500–2. 10.1097/MPG.0b013e3180691168 18030225

[5] Walco GA Cassidy RC Schechter NL. Pain, hurt, and harm. The ethics of pain control in infants and children. N Engl J Med. (1994) 331:541–4. 10.1056/NEJM199408253310812 8041423

[6] Lightdale JR Valim C Mahoney LB. Sharon Wong null, DiNardo J, Goldmann DA. Agitation during procedural sedation and analgesia in children. Clin Pediatr. (2010) 49:35–42. 10.1177/0009922809344425 19738182

[7] Andrade S Battistuz E Di Leo G Barbi E. Propofol as standard of care for pediatric sedation for short procedures, such as upper endoscopy. J Pediatr Gastroenterol Nutr. (2020) 70:e52. 10.1097/MPG.0000000000002561 31978037

[8] Yang SM Yi DY Choi GJ Lim IS Chae SA Yun SW. effects of sedation performed by an anesthesiologist on pediatric endoscopy: a single-center retrospective study in Korea. J Korean Med Sci. (2020) 35:e183. 10.3346/jkms.2020.35.e183 32476304 PMC7261697

[9] Amornyotin S Aanpreung P. Clinical effectiveness of an anesthesiologist-administered intravenous sedation outside of the main operating room for pediatric upper gastrointestinal endoscopy in Thailand. Int J Pediatr. 2010;2010. 10.1155/2010/748564 PMC2929513 20811603

[10] Jagadisan B A. survey of procedural sedation for pediatric gastrointestinal endoscopy in India. Indian J Gastroenterol Off. (2015) 34:158–63. 10.1007/s12664-015-0556-5 25917522

[11] Acquaviva MA Horn ND Gupta SK. Endotracheal intubation versus laryngeal mask airway for esophagogastroduodenoscopy in children. J Pediatr Gastroenterol Nutr. (2014) 59:54–6. 10.1097/MPG.0000000000000348 24637966

[12] van Beek EJAH Leroy PLJM. Safe and effective procedural sedation for gastrointestinal endoscopy in children. J Pediatr Gastroenterol Nutr. (2012) 54:171–85. 10.1097/MPG.0b013e31823a2985 21975965

[13] Khoshoo V Thoppil D Landry L Brown S Ross G. Propofol versus midazolam plus meperidine for sedation during ambulatory esophagogastroduodenoscopy. J Pediatr Gastroenterol Nutr. (2003) 37:146–9. 10.1097/00005176-200308000-00012 12883300

[14] Kaddu R Bhattacharya D Metriyakool K Thomas R Tolia V. Propofol compared with general anesthesia for pediatric GI endoscopy: is propofol better? Gastrointest Endosc. (2002) 55:27–32. 10.1067/mge.2002.120386 11756910

[15] Disma N Astuto M Rizzo G Rosano G Naso P Aprile G. Propofol sedation with fentanyl or midazolam during oesophagogastroduodenoscopy in children. Eur J Anaesthesiol. (2005) 22:848–52. 10.1017/S0265021505001432 16225720

[16] Elitsur Y Blankenship P Lawrence Z. Propofol sedation for endoscopic procedures in children. Endoscopy. (2000) 32:788–91. 10.1055/s-2000-7713 11068839

[17] Sheahan CG Mathews DM. Monitoring and delivery of sedation. Br J Anaesth. (2014) 113 Suppl 2:ii37–47. 10.1093/bja/aeu378 25498581

[18] Lightdale JR Acosta R Shergill AK Chandrasekhara V Chathadi K Early D. Modifications in endoscopic practice for pediatric patients. Gastrointest Endosc. (2014) 79:699–710. 10.1016/j.gie.2013.08.014 24593951

[19] Biber JL Allareddy V Allareddy V Gallagher SM Couloures KG Speicher DG. Prevalence and predictors of adverse events during procedural sedation anesthesia-outside the operating room for esophagogastroduodenoscopy and colonoscopy in children: age is an independent predictor of outcomes. Pediatr Crit Care Med. (2015) 16:e251–259. 10.1097/PCC.0000000000000504 26218257

[20] Lightdale JR Liu QY Sahn B Troendle DM Thomson M Fishman DS. Pediatric endoscopy and high-risk patients: a clinical report from the NASPGHAN endoscopy committee. J Pediatr Gastroenterol Nutr. (2019) 68:595–606. 10.1097/MPG.0000000000002277 30664560 PMC8597353

[21] Thakkar K El-Serag HB Mattek N Gilger MA. Complications of pediatric EGD: a 4-year experience in PEDS-CORI. Gastrointest Endosc. (2007) 65:213–21. 10.1016/j.gie.2006.03.015 17258979

[22] Thakkar K El-Serag HB Mattek N Gilger M. Complications of pediatric colonoscopy: a five-year multicenter experience. Clin Gastroenterol Hepatol Off. (2008) 6:515–20. 10.1016/j.cgh.2008.01.007 PMC4999080 18356115

[23] Lightdale JR Valim C Newburg AR Mahoney LB Zgleszewski S Fox VL. Efficiency of propofol versus midazolam and fentanyl sedation at a pediatric teaching hospital: a prospective study. Gastrointest Endosc. (2008) 67:1067–75. 10.1016/j.gie.2007.11.038 18367187

[24] Helsø I Risom M Vestergaard TR Foss NB Rosenstock S Møller MH. Anaesthesia in patients undergoing esophago-gastro-duodenoscopy for suspected bleeding. Dan Med J. (2017) 64:A5409. Available online at: https://ugeskriftet.dk/dmj/anaesthesia-patients-undergoing-esophago-gastro-duodenoscopy-suspected-bleeding 28975884

[25] Coté CJ Wilson S. Guidelines for monitoring and management of pediatric patients before, during, and after sedation for diagnostic and therapeutic procedures. Pediatrics. (2019) 143:26E–52E. 10.1542/peds.2019-1000 31439094

[26] Najafi N Veyckemans F Vanhonacker D Legrand C Van de Velde A Vandenplas Y. Incidence and risk factors for adverse events during monitored anaesthesia care for gastrointestinal endoscopy in children: a prospective observational study. Eur J Anaesthesiol. (2019) 36:390–9. 10.1097/EJA.0000000000000995 30950900

[27] Liacouras CA Mascarenhas M Poon C Wenner WJ. Placebo-controlled trial assessing the use of oral midazolam as a premedication to conscious sedation for pediatric endoscopy. Gastrointest Endosc. (1998) 47:455–60. 10.1016/s0016-5107(98)70244-5 9647368

[28] ASA Physical Status Classification System. (2021). Available online at: https://www.asahq.org/standards-and-guidelines/asa-physical-status-classification-system. (accessed May 13, 2021).

[29] Continuum of Depth of Sedation: Definition of General Anesthesia and Levels of Sedation/Analgesia. (2021). Available online at: https://www.asahq.org/standards-and-guidelines/continuum-of-depth-of-sedation-definition-of-general-anesthesia-and-levels-of-sedationanalgesia (accessed May 11, 2021).

[30] Agostoni M Fanti L Gemma M Pasculli N Beretta L Testoni PA. Adverse events during monitored anesthesia care for GI endoscopy: an 8-year experience. Gastrointest Endosc. (2011) 74:266–75. 10.1016/j.gie.2011.04.028 21704990

[31] Mamula P Markowitz JE Neiswender K Zimmerman A Wood S Garofolo M. Safety of intravenous midazolam and fentanyl for pediatric GI endoscopy: prospective study of 1578 endoscopies. Gastrointest Endosc. (2007) 65:203–10. 10.1016/j.gie.2006.05.002 17258977

[32] Conway A Rolley J Sutherland JR. Midazolam for sedation before procedures. Cochrane Database Syst Rev. (2016) (5):CD009491. 10.1002/14651858.CD009491.pub2 PMC6517181 27198122

[33] Wennberg JE. Unwarranted variations in healthcare delivery: implications for academic medical centres. BMJ. (2002) 325:961–4. 10.1136/bmj.325.7370.961 12399352 PMC1124450

[34] Taenzer AH Sites BD Kluger R Barrington M. Settled science or unwarranted variation in local anesthetic dosing? An analysis from an International Registry of Regional Anesthesiology. Reg Anesth Pain Med. (2019) 44:998–1002. 10.1136/rapm-2019-100650 31494594

[35] Cook DJ Pulido JN Thompson JE Dearani JA Ritter MJ Hanson AC. Standardized practice design with electronic support mechanisms for surgical process improvement: reducing mechanical ventilation time. Ann Surg. (2014) 260:1011–5. 10.1097/SLA.0000000000000726 24836149

[36] Rajasekaran S Hackbarth RM Davis AT Kopec JS Cloney DL Fitzgerald RK. The safety of propofol sedation for elective nonintubated esophagogastroduodenoscopy in pediatric patients. Pediatr Crit Care Med. (2014) 15:e261–269. 10.1097/PCC.0000000000000147 24849145

[37] Cohen S Glatstein MM Scolnik D Rom L Yaron A Otremski S. Propofol for pediatric colonoscopy: the experience of a large, tertiary care pediatric hospital. Am J Ther. (2014) 21:509–11. 10.1097/MJT.0b013e31826a94e9 23567786

[38] Hsieh YH Chou AL Lai YY Chen BS Sia SL Chen IC. Propofol alone versus propofol in combination with meperidine for sedation during colonoscopy. J Clin Gastroenterol. (2009) 43:753–7. 10.1097/MCG.0b013e3181862a8c 19169146

[39] Narula N Masood S Shojaee S McGuinness B Sabeti S Buchan A. Safety of propofol versus nonpropofol-based sedation in children undergoing gastrointestinal endoscopy: a systematic review and meta-analysis. Gastroenterol Res Pract. (2018) 2018:6501215. 10.1155/2018/6501215 30210535 PMC6126059

[40] Milius EM Papademetrious TR Heitlinger LA. Retrospective review of propofol dosing for procedural sedation in pediatric patients. J Pediatr Pharmacol Ther. (2012) 17:246–51. 10.5863/1551-6776-17.3.246 23258967 PMC3526928

